# Supplementary material for: The Transcription Factor MbWRKY46 in Malus baccata (L.) Borkh Mediate Cold and Drought Stress Responses
Source: Int J Mol Sci. 2023 Aug 5;24(15):12468. doi: 10.3390/ijms241512468 (PMC10420220; doi:10.3390/ijms241512468)
Supplement: Supplementary file 1 [file ijms-24-12468-s001.zip › ijms-2502283-supplementary.pdf]

Table S1 List of primers used in this study.

Primer Name	PrimerSequence (5'→3')	Purpose
MdWRKY46-F	ATGGATTCTAGTAAGAGCTGGG	full-length cDNA of *MdWRKY46*
MdWRKY46-R	TTATAAGAAAAATCCTGGGCA	full-length cDNA of *MdWRKY46*
MdWRKY46-2F	GAGCTCGGTACCCGGGGATCCATGGATTCTAGTAAGAGCTGGG	subcellular localization
MdWRKY46-2R	GCCCTTGCTCACCATGTCGACTAAGAAAAATCCTGGGCAGTC	subcellular localization
MdWRKY46-qF	CAAGCCAGATGAGAAACA	qPCR
MdWRKY46-qR	TCAAACGGGAAATAGGAG	qPCR
Actin-F	ACACGGGGAGGTAGTGACAA	qPCR
Actin-R	CCTCCAATGGATCCTCGTTA	qPCR
AtKIN1-F	AGCACAACAGGCGGGAAAG	qPCR
AtKIN1-R	GTGGTGGTTCCTCTGTTTGATCCA	qPCR
AtRD29A-F	GTGCCGACGGGATTTGACG	qPCR
AtRD29A-R	CCCCAAGAAATCAAACAA	qPCR
AtCOR47A-F	ACAGAGGAATCACCAGCGACG	qPCR
AtCOR47A-R	CGTTGTTCTTGTACTCCTCAGCCAT	qPCR
AtDREB2A-F	TGGAGAATGGTGCGGAAGA	qPCR
AtDREB2A-R	CTCCACTCTGATCATAAACTGCCAT	qPCR
AtERD10-F	AACACCGTTCCAGAGCAGG	qPCR
AtERD10-R	CGGTGTTCTTGTACTCTTCTGCCAT	qPCR
AtRD29B-F	CGGTGTTCTTGTACTCTTCTGCCAT	qPCR
AtRD29B-R	GAAGGAGACGCAACAAGGG	qPCR
MdWRKY46-hF	GAGCTCGGTACCCGGGGATCCATGGATTCTAGTAAGAGCTGGG	homologous recombination
MdWRKY46-hR	TGCCTGCAGGTCGACTCTAGATAAGAAAAATCCTGGGCAGTC	homologous recombination

Table S2. The PCR reaction system of *MbWRKY46*

Component	Volume
cDNA	1.5 μL
MbWRKY46-F	1.0 μL
MbWRKY46-R	1.0 μL
2×Easy Taq® PCR SuperMix	12.5 μL
dd H_2O	9.0 μL
Total Volume	25 μL

Table S3. PCR reaction conditions

Step	Temperature	Time
Pre denaturation	95 ℃	5 min
Denaturation	95 ℃	45 s
Annealing	55 ℃	1 min
Extend	72℃	5 min
