# Supplementary figures and images for: The Transcription Factor MbWRKY46 in Malus baccata (L.) Borkh Mediate Cold and Drought Stress Responses
Source: Int J Mol Sci. 2023 Aug 5;24(15):12468. doi: 10.3390/ijms241512468 (PMC10420220; doi:10.3390/ijms241512468)

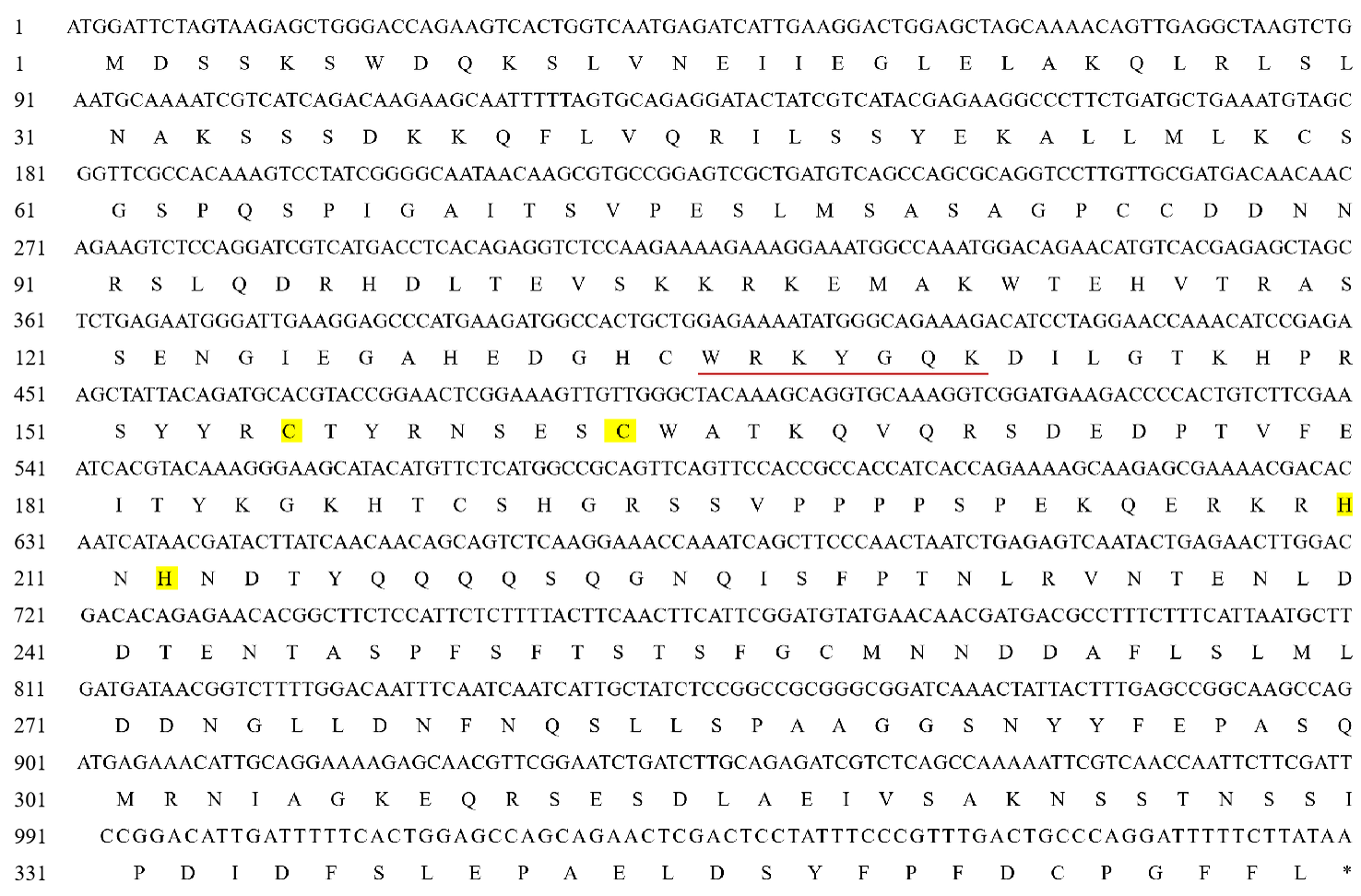

Supplement: Supplementary file 1 [file ijms-24-12468-s001.zip › Figgure S1.tif]
